# Clinical nurse’s knowledge, attitude, and practice regarding the Intrinsic Capacity of the aged: A cross-sectional study

**DOI:** 10.1371/journal.pone.0330471

**Published:** 2026-03-19

**Authors:** Yi Ren, Xiumin Zhang

**Affiliations:** Nursing Department, People’s Hospital of Xinjiang Uygur Autonomous Region, XinJiang, China; Erasmus University Rotterdam, NETHERLANDS, KINGDOM OF THE

## Abstract

**Background:**

Within the context of Chinese healthcare settings, from the perspective of clinical nursing practice in China, the present study was designed to systematically evaluate registered nurses’ theoretical knowledge, attitude, and practice(KAP)toward assessing the Intrinsic Capacity in the aging population.

**Methods:**

This cross-sectional study based on online questionnaires included 606 clinical nurses who were employed in a tertiary hospital in Xinjiang from November 21, 2024 to February 18, 2025. Using the Delphi method to create a self-made online questionnaire to collect participants’ sociodemographic information and KAP scores of changes in the Intrinsic Capacity of the elderly.

**Results:**

Of the collected data, 606 questionnaires were deemed valid for analysis. the research instrument showed high reliability, as evidenced by Cronbach’s α values of 0.979 for knowledge, 0.916 for attitude, and 0.936 for practice sections. Exploratory factor analysis was conducted, and the Bartlett’s test of sphericity result was 0.814.The mean knowledge score was 34.75 ± 11.165(possible range:18–90).The mean attitude score was 20.86 ± 5.488(possible range: 11–35).The mean practice score was 32.69 ± 8.695(possible range:16–72). Knowledge, attitude, and practice (KAP) scores of clinical nurses regarding the intrinsic capacity of older patients were treated as dependent variables. LASSO regression was employed to identify significant predictors. The results indicated that: nursing hierarchy (*λ* = 0.509), prior receipt of intrinsic capacity training (*λ* = 1.739), hospital support for new technology development in geriatric care (*λ* = 2.919), and nurses’ perceived adequacy of existing knowledge to meet clinical needs (*λ* = 4.755) were independently associated with knowledge scores. For attitude scores, significant predictors included hospital support for new technology development in geriatric care (*λ* = 1.846), perceived adequacy of existing knowledge to meet clinical needs (*λ* = 1.580), and expected frequency of training (*λ* = 0.699). Practice scores were independently associated with prior receipt of intrinsic capacity training (*λ* = 1.914), hospital support for new technology development in geriatric care (*λ* = 0.144), the proportion of patients aged over 60 cared for during the past year (*λ* = 1.176), and perceived adequacy of existing knowledge to meet clinical needs (*λ* = 2.696). The structural equation modeling path coefficients revealed significant direct pathways: from knowledge to attitude(*r* = 0.732, *P* < 0.01), knowledge to practice(*r* = 0.617, *P* < 0.01), and attitude to practice(*r* = 0.666, *P* < 0.01).The bootstrap mediation effect test results indicated an indirect effect (*B* = 0.421, *95%CI*: 0.296–0.558) and a direct effect (*B* = 0.295, *95%CI*: 0.146–0.451), with effect proportions of 59% and 41%, respectively.

**Conclusion:**

This investigation reveals the insufficient understanding, attitude, and practice of clinical nurses in Xinjiang towards changes in the Intrinsic Capacity of elderly patients.

## 1. Introduction

Population aging is a dynamic demographic process characterized by a gradual decrease in the proportion of young people and a corresponding increase in the proportion of elderly individuals, resulting in a higher overall aging population. In China, this demographic trend is expected to persist over the long term, representing a fundamental national condition [1]. Globally, the degree of population aging is steadily increasing, and as the world’s most populous country, China faces particularly severe aging challenges. According to the 2022 National Population Change Survey conducted by the National Bureau of Statistics [2], individuals aged 60 years and above constitute 19.8% of the total population, and those aged 65 years and above account for 14.9%, positioning China among the most rapidly aging nations worldwide. Moreover, a report on the path to healthy aging in China [3] highlighted that life expectancy has steadily increased, approaching levels observed in developed countries. Those born during the second wave of the baby boom (1962–1975) began entering retirement in 2022, further exacerbating the aging burden in China.

In 2015, the World Health Organization (WHO) released the World Report on Aging and Health [4], introducing the evidence-based concept of healthy aging to guide global strategies. Healthy aging does not imply the absence of disease or functional decline but rather emphasizes the preservation and optimization of functional abilities necessary to maintain quality of life in older age [4]. A core component of this concept is Intrinsic Capacity (IC), which refers to the composite of an individual’s physical and mental capacities that can be mobilized at any time and forms the basis of functional ability [5]. This paradigm shift from a disease-centered to a function-centered medical model promotes proactive, personalized, and multidisciplinary interventions aimed at maintaining IC, extending healthy life expectancy, and delaying disability. The introduction of IC not only informs global aging strategies but also underpins the United Nations Decade of Healthy Aging (2021–2030) initiatives.

Recently, with the acceleration of global population aging, the concept of IC has gained recognition among clinicians and researchers [6–9]. Increasing evidence indicates that age-related biological changes can simultaneously influence the prognosis of multiple chronic conditions [10–11]. However, research on IC in China remains at an early stage, primarily focusing on community-dwelling older adults, with limited studies addressing hospitalized elderly patients [12–14]. In 2021, the WHO proposed the Ten-Year Action Plan for Healthy Aging 2020–2030 [15], emphasizing the need to change perceptions and behaviors regarding aging and to support active interventions to maintain IC. Despite awareness of global aging trends, clinical medical staff often concentrate on disease diagnosis rather than the functional decline associated with reduced IC, likely reflecting insufficient knowledge, attitudes, and practices regarding the impact of IC decline on disease management and prognosis [16]. Such gaps may constrain the effectiveness of healthy aging frameworks and blur distinctions between IC and related concepts such as frailty, biological aging, geriatric science, and geriatric syndromes.

Evidence indicates that IC represents a dynamic construct reflecting functional trajectories throughout the life course [17]. Research further suggests that prioritizing IC in older adults may be more consequential than focusing solely on individual chronic diseases [18]. As a multidimensional evaluation model, IC captures physical reserve capacities, emphasizes positive health attributes, and serves as a critical predictor of adverse health outcomes [6]. Early identification of individuals at risk of functional decline facilitates personalized clinical and public health interventions, tailored to age, comorbidities, and patient preferences. Implementing individualized care plans can potentially prevent, slow, or reverse declines in IC, underscoring the importance of accurate knowledge and positive attitudes among clinical nurses.

The Knowledge, Attitude, and Practice (KAP) framework provides a dual-purpose research tool for capturing both quantitative and qualitative insights into cognitive gaps, misconceptions, and interpretive inaccuracies within targeted populations [19,20]. Accordingly, this study aims to assess the KAP of nursing staff regarding declines in IC among older adults, identify populations in urgent need of educational interventions, and inform strategies to enhance nurses’ knowledge, attitudes, and practices in clinical care.

## 2. Materials and methods

### 2.1. Research design and research object

This cross-sectional study was conducted from November 21, 2024, to February 18, 2025, including on-duty clinical nurses from a tertiary hospital in Xinjiang. A total of 634 questionnaires were collected. After excluding 18 questionnaires with patterned responses or logical inconsistencies and 10 questionnaires in which respondents selected “uncertain” for all continuous variables, 606 valid responses were retained for final analysis. These included the initial 50 pilot responses that had been used for reliability and validity testing of the instrument.Due to the distribution method, the precise response rate could not be determined.

The study protocol was approved by the Ethics Committee of the People’s Hospital of Xinjiang Uygur Autonomous Region (approval number KY2024111501). All participants provided informed consent after receiving a detailed explanation of the study objectives and procedures.

To assess representativeness, the demographic characteristics of respondents were compared with the overall nursing staff in the hospital. The sample was predominantly female (95.2%), aged 26–35 years (52.8%), with 2–10 years of service (37.6%), holding an undergraduate degree (70.8%), and occupying nurse professional titles (33.3%). Compared with the hospital nursing workforce, these distributions are consistent with overall averages, suggesting that the sample is generally representative of the hospital’s clinical nursing staff.

### 2.2. Questionnaire

The design of the questionnaire was based on the 2015 WHO Guidelines: Integrated Care for Older People(ICOPE): guidance for person-centred ass essment and pathways in primary care [5] and the related literature [4,14,21–33]. The research team searched China National Knowledge Infrastructure, Wanfang Medical Network, and Baidu Scholar,PubMed,Web of Science,A review was conducted on the literature related to the knowledge, attitudes, and practices of clinical nurses regarding the internal abilities of elderly patients, with reference to global and domestic guidelines and policies on aging. Through brainstorming by the research group, a questionnaire item pool was formed for clinical nurses regarding the knowledge, attitudes, and practices of elderly patients regarding their internal abilities.Using the Delphi method, divide the inquiry questionnaire into two parts. Letter to experts: including the topic for filling out the form and expert rating form: The expert rating form adopts a combination of closed ended and open-ended questions. Experts evaluate whether the listed items belong to the corresponding dimensions and rate the importance of each item one by one. The Likert 5-point rating method is used, ranging from “very unimportant” to “very important”, with a score of 1–5 points. Each item is accompanied by a modification suggestion column and a new item column. Experts can provide opinions or delete or supplement items. The expert group has selected 15 qualified nursing experts, all of whom hold a master’s degree or above, have been engaged in nursing work for at least 10 years, have relevant clinical experience in elderly care, and have an understanding of the concept of Intrinsic Capacity.Two rounds of expert consultation were conducted. In total, 15 questionnaires were distributed in each round, with all 30 returned, yielding a 100% response rate (expert engagement coefficient). The expert authority coefficients for the two rounds were 0.747 and 0.793, respectively. The mean importance ratings of questionnaire items ranged from 3.267 to 4.933 in the first round and from 4.267 to 5.000 in the second round. The coefficients of variation ranged from 0.052 to 0.244 and from 0.000 to 0.187, respectively. Kendall’s coefficients of concordance were 0.457 in the first round and 0.533 in the second round (both P < 0.05), indicating improved expert consensus. In the first round, experts recommended the addition of 10 items and the removal of 4 items, which were incorporated. No further objections were raised in the second round. Following the two rounds of consultation, the Questionnaire on Knowledge, Attitudes, and Practices of Clinical Nurses Regarding the Intrinsic Capacity of Older Patients was finalized.

The questionnaire was piloted on 50 nurses using the questionnaire to evaluate the overall suitability, understandability and clarity of the instrument for the participants.The validated Chinese version of the research instrument contained four systematically designed sections:(1) Including age, gender, years of work experience, education level, nursing title, clinical position, and elderly care experience,(2) knowledge dimension,(3) attitude dimension, and(4) practice dimension.Of the collected data, 606 questionnaires were deemed valid for analysis. The research instrument showed high reliability, as evidenced by Cronbach’s α values of 0.979 for knowledge, 0.916 for attitude, and 0.936 for practice sections. The pilot study’s face validity assessment revealed no problematic items, as participants reported no comprehension difficulties. Exploratory factor analysis was conducted, and the Bartlett’s test of sphericity result was 0.814.

The scale was finally formed into a Chinese version of the online questionnaire. For example, the professional titles of nurses are classified as: nurse, nurse in charge, deputy chief nurse and chief nurse. The levels of obtaining specialist nurse certificates include: hospital level of medical institutions, autonomous region or provincial level and national level. The knowledge construct(k) was operationalized through a 20-item scale, with cumulative scores potentially ranging from 20 to 100 points.It uses a 5-point Likert scale, in which 1 represents the answer of “strongly agree”, 2 represents “agree”, 3 represents “uncertain”, “4 represents disagree”, and 5 represents very disagree “. This study’s knowledge(K) assessment encompasses five key aspects of intrinsic capacity in older adults: conceptualization of intrinsic capacity(items 1–2), measurement protocols(items 3–6), predisposing factors(items 7–9), screening mechanisms(items 10–14), and therapeutic interventions(items 15–20) relevant to geriatric care.The attitude(A) assessmentcomprises 11 items measured on a 5-point Likert scale(1 = strongly agree to 5 = strongly disagree), yielding total scores between 11 and 55 points. Similarly, the practice(P) evaluation incorporates 16 items using the same 5-point Likert scale, with potential total scores ranging from 16 to 90 points.

### 2.3. Data collection methodology and quality control mechanisms

The research team developed an online survey instrument through the Questionnaire Star platform, subsequently creating a QR code for WeChat-based data collection. Study participants accessed the questionnaire by scanning the provided QR code and completed the survey electronically. With the aim of guaranteeing the methodological rigor and data accuracy of the research instrument,the home page of the questionnaire star explains the purpose of the survey and informed consent. The respondents can enter the questionnaire answering session after they know and consent. If participants encounter any questions when answering, the members of the research group are responsible for interpreting and solving the problems.Subsequent to data collection, a comprehensive quality assessment was conducted, with exclusion criteria applied to questionnaires exhibiting response patterns or logical errors, thereby ensuring data integrity.

## 3. Data analysis approach

According to epidemiological survey sampling guidelines and cross-sectional study sample size calculation methods [34], a reported incidence rate of impaired intrinsic capacity among older adults is 57.7% (13). Based on this, a minimum of 470 respondents were required to ensure statistical validity in this study. After accounting for an estimated 20% rate of invalid questionnaires, the target sample size was adjusted to 588 participants. All questionnaires were administered anonymously to reduce social desirability bias. Participants were instructed not to include any personally identifying information, and responses were collected and stored confidentially. This approach aimed to encourage honest and accurate reporting of knowledge, attitudes, and practices regarding Intrinsic Capacity.

All statistical analyses were performed using IBM SPSS Statistics version 25.0 (Armonk, NY, USA).Continuous variables were expressed as mean ± standard deviation (mean ± SD). The normality of continuous data was confirmed by Shapiro-Wilk test (p > 0.05), and homogeneity of variance was verified by Levene’s test (p > 0.05). These variables were analyzed using Student’s t-tests or analysis of variance (ANOVA). Categorical variables were presented as frequencies and percentages [n (%)]. All contingency table cells had expected frequencies >5, meeting the applicability conditions for Pearson’s chi-square test, which was employed for evaluation.Pearson’s correlation method was used to assess associations among KAP components. To analyze factors influencing nurses’ knowledge, attitudes, and practices (KAP) regarding Intrinsic Capacity (IC), as well as IC-related scores, both univariate and multivariate linear stepwise regression analyses were conducted. The dependent variables were the aggregated IC scores, calculated by summing the responses across all items within each KAP domain (knowledge, attitude, and practice), with higher scores indicating greater knowledge, more positive attitudes, or better practice behaviors.Independent variables included demographic and professional characteristics of nurses (age, sex, years of service, educational background, professional title, and nursing level), as well as exposure to IC-related training, participation in geriatric care programs, hospital support for geriatric innovation, and nurses’ self-reported perception of whether their current IC knowledge meets clinical needs. Variables showing statistical significance (*P* < 0.05) in univariate analysis were entered into multivariate stepwise regression models to identify independent factors associated with IC scores.This approach provides a replicable framework for examining which demographic, professional, and organizational factors are significantly related to nurses’ KAP scores on Intrinsic Capacity.Structural equation modeling was conducted using AMOS in IBM SPSS Statistics version 25.0 (Armonk, NY, USA) to examine three hypothesized relationships: the direct effect of knowledge on attitude (a), the direct effect of knowledge on practice (c), and the indirect effect of knowledge on practice mediated by attitude (b). A two-tailed p-value threshold of < 0.05 was established for determining statistical significance.

### 3.1. Data handling

Of the 634 questionnaires initially collected, 28 were excluded due to patterned responses, logical inconsistencies, or the selection of “uncertain” for all continuous variables. Consequently, 606 valid questionnaires were retained for analysis. Because the study employed an online survey platform with mandatory response settings, no missing values were present in the dataset. All statistical analyses were therefore conducted on a complete case basis. The potential impact of excluding patterned or inconsistent responses on the generalizability of the findings is acknowledged as a methodological limitation and addressed in the Discussion section.

## 4. Results

Prior to formal data collection, a pilot survey of 50 questionnaires was conducted to evaluate the reliability and validity of the instrument. Cronbach’s α values were 0.979 for the knowledge dimension, 0.916 for attitude, and 0.936 for practice, all indicating excellent internal consistency. The Kaiser–Meyer–Olkin measure of sampling adequacy was 0.814, and Bartlett’s test of sphericity was statistically significant (*P* < 0.001), confirming the appropriateness of factor analysis for the scale.

Among participants, 95.2% were female, 52.8% were aged 26–35 years, 37.6% had 2–10 years of service, 70.8% held an undergraduate degree, 33.3% had a nurse professional title, 32.5% were at N2 level, and 74.3% worked in tertiary hospitals (Table 1).

**Table 1:** Characteristics and KAP scores of the participant.

Variable	N(%)	Knowledge	*t*/*F*	LSD	Attitude	*t*/*F*	LSD	Practice	*t*/*F*	LSD
Gender										
Male	29(4.80%)	37.90 ± 15.467	1.555	/	21.97 ± 6.339	1.116	/	34.45 ± 11.012	1.116	/
Female	577(95.20%)	34.60 ± 10.899			20.80 ± 5.442			32.60 ± 8.565		
Age										
≤25years	99(16.30%)	32.72 ± 9.037	3.017**	①③,②③	20.28 ± 5.087	0.853	/	31.87 ± 8.024	0.637	/
26-35years	320(52.80%)	34.30 ± 11.578			20.76 ± 5.647			32.59 ± 9.258		
36-45years	153(25.20%)	36.57 ± 11.371			21.31 ± 5.637			33.22 ± 8.190		
≥46years	34(5.60%)	36.76 ± 10.849			21.41 ± 4.279			33.65 ± 7.278		
Seniority										
≤2years	102(16.80%)	32.54 ± 9.439	3.157*	①③,①④,②④	19.87 ± 5.094	1.059	/	31.59 ± 8.714	0.811	/
2-10years	228(37.60%)	33.97 ± 10.775			20.92 ± 5.249			32.48 ± 8.080		
11-20years	206(34.00%)	35.64 ± 12.137			21.17 ± 5.952			33.33 ± 9.566		
21-30years	59(9.70%)	38.27 ± 11.135			21.20 ± 5.586			32.86 ± 7.791		
≥31years	11(1.80%)	36.09 ± 10.625			20.91 ± 3.833			34.18 ± 8.518		
Education										
Secondary specialized school	3(0.50%)	25.00 ± 11.269	3.398*	②③	19.33 ± 5.132	0.548	/	31.00 ± 9.849	0.792	/
Junior college	171(28.20%)	32.79 ± 10.373			20.47 ± 5.382			31.91 ± 8.931		
Undergraduate	429(70.80%)	35.59 ± 11.400			21.01 ± 5.552			33.03 ± 8.619		
Graduate and above	3(0.50%)	36.33 ± 0.577			22.33 ± 0.577			30.33 ± 2.082		
Professional title										
Nurse	171(28.20%)	32.97 ± 9.745	3.348**	①③,①④,②④	20.33 ± 4.931	1.000	/	31.60 ± 8.311	1.199	/
Junior nurse	202(33.30%)	34.38 ± 10.938			21.33 ± 5.795			33.35 ± 8.396		
Intermediate nurse	200(33.00%)	35.83 ± 12.262			20.71 ± 5.671			32.81 ± 9.369		
Associate chief nurse	28(4.60%)	40.04 ± 10.62			21.75 ± 5.345			33.11 ± 7.187		
chief superintendent nurse	5(0.90%)	38.20 ± 12.478			20.80 ± 3.347			36.20 ± 12.276		
Nursing level										
Ungraded	24(4.00%)	33.63 ± 8.627	2.683*	②⑤,②⑥, ③⑥,④⑥	19.63 ± 4.924	0.915	/	32.58 ± 10.172	0.761	/
N0	73(12.00%)	32.00 ± 9.497			19.90 ± 5.162			31.19 ± 8.798		
N1	111(18.30%)	34.28 ± 10.439			21.11 ± 4.842			32.18 ± 7.837		
N2	197(32.50%)	34.27 ± 11.329			20.99 ± 5.762			33.03 ± 8.691		
N3	165(27.20%)	36.07 ± 11.781			21.21 ± 5.888			33.04 ± 8.939		
N4 and above	36(5.90%)	39.19 ± 12.723			20.50 ± 4.884			33.89 ± 8.998		
Position										
No position	474(78.20%)	34.76 ± 10.913	1.074	/	21.00 ± 5.546	0.708	/	32.66 ± 8.573	1.483	/
Total teaching	19(3.10%)	34.32 ± 16.981			19.89 ± 6.136			32.79 ± 13.336		
Nursing team leader	29(4.80%)	33.14 ± 11.413			19.52 ± 5.572			29.41 ± 8.894		
Head nurse	48(7.90%)	37.33 ± 11.681			20.52 ± 4.855			34.13 ± 7.396		
Other	36(5.90%)	32.81 ± 9.718			21.00 ± 5.160			33.69 ± 8.522		
Do you have a specialized certificate										
Yes	275(45.40%)	34.30 ± 11.380	−0.909	/	20.40 ± 5.386	−1.870	/	31.83 ± 8.593	−2.228*	/
No	331(54.60%)	35.13 ± 10.988			21.24 ± 5.551			33.40 ± 8.727		
Specialized certificate level										
Hospital level	90(14.90%)	33.47 ± 11.655	2.480	/	20.20 ± 5.597	2.110	/	31.71 ± 8.283	2.236	/
Autonomous Region Level	101(16.70%)	34.24 ± 11.688			20.45 ± 5.465			32.33 ± 8.706		
National level	122(20.10%)	33.18 ± 10.477			20.30 ± 5.214			31.53 ± 8.319		
Not obtained	293(48.30%)	35.98 ± 11.022			21.43 ± 5.541			33.60 ± 8.909		
There is a father or mother who is over 60 years old at home										
Yes	369(60.90%)	35.25 ± 11.762	1.371	/	20.95 ± 5.627	0.515	/	33.19 ± 9.115	1.812	/
No	237(39.10%)	33.98 ± 10.143			20.71 ± 5.272			31.92 ± 7.954		
Hospital level										
Secondary hospital	152(25.10%)	34.00 ± 10.322	0.632	/	20.75 ± 5.034	0.226	/	33.02 ± 8.120	0.373	/
Tertiary hospital	450(74.30%)	34.98 ± 11.477			20.88 ± 5.653			32.55 ± 8.904		
Below level 2	4(0.70%)	38.25 ± 3.304			22.00 ± 3.559			35.50 ± 6.455		
Received training on internal ability										
Yes	341(56.30%)	32.44 ± 10.363	−5.958***	/	20.06 ± 5.324	−4.113***	/	31.06 ± 8.518	−5.337***	/
No	265(43.70%)	37.74 ± 11.468			21.88 ± 5.534			34.78 ± 8.486		
Participated in geriatric nursing related training										
Yes	299(49.30%)	32.04 ± 10.487	−6.070***	/	19.89 ± 5.187	−4.358***	/	31.13 ± 8.500	−4.433***	/
No	307(50.70%)	37.39 ± 11.190			21.80 ± 5.615			34.21 ± 8.626		
The hospital supported the development of new technology of geriatric nursing										
Yes	463(76.40%)	33.40 ± 10.863	−5.521***	/	20.22 ± 5.260	−5.265***	/	31.73 ± 8.475	−4.973***	/
No	143(23.60%)	39.15 ± 11.032			22.92 ± 5.715			35.79 ± 8.702		
Proportion of patients aged 60 and above who have been cared for during clinical work in the past year										
0-25%	45(7.40%)	35.76 ± 10.512	1.241	/	20.53 ± 3.739	0.719	/	35.16 ± 7.862	3.426*	①④,②④
26-50%	106(17.50%)	35.08 ± 11.241			21.20 ± 4.964			34.08 ± 8.417		
51-75%	237(39.10%)	35.47 ± 11.635			21.10 ± 5.897			32.65 ± 8.963		
76-100%	218(36.00%)	33.61 ± 10.708			20.49 ± 5.578			31.54 ± 8.548		
The current knowledge of internal ability can meet the needs of daily clinical work										
Completely satisfied	83(13.70%)	25.24 ± 10.077	33.222***	①②, ①③,①④, ②③, ②④	17.40 ± 5.233	16.012***	①②, ①③, ①④,②③,②④	28.29 ± 9.345	13.044***	①③, ①④,②③, ②④,③④
Mostly satisfied	189(31.20%)	32.34 ± 10.095			19.85 ± 5.064			30.93 ± 8.483		
Basically satisfied	236(38.90%)	37.17 ± 9.467			22.18 ± 5.153			33.75 ± 7.181		
Not satisfied	93(15.30%)	40.69 ± 9.964			22.57 ± 5.549			36.75 ± 8.745		
Not satisfied at all	5(0.80%)	59.60 ± 18.703			22.00 ± 7.071			46.60 ± 15.291		
The expected frequency of receiving training on internal ability										
Every month	86(14.20%)	29.98 ± 11.326	7.020***	①②, ①③,①④	18.80 ± 5.163	7.396***	①②, ①③, ①④, ②④	30.06 ± 8.305	3.466*	①②, ①③, ①④
Quarterly	236(38.90%)	34.87 ± 10.319			20.56 ± 5.122			33.05 ± 8.838		
Every half year	137(22.60%)	36.61 ± 11.074			21.31 ± 5.295			33.74 ± 8.584		
annually	147(24.30%)	35.63 ± 11.783			22.12 ± 6.041			32.67 ± 8.571		

* Correlation is significant at the 0.05 level(2-tailed); ** Correlation is significant at the 0.01 level(2-tailed); *** Correlation is significant at the 0.001 level(2-tailed).

The mean knowledge score was 34.75 ± 11.17 (range: 18–90). Higher knowledge scores were associated with being over 35 years of age (*P <* 0.01), longer work experience (*P <* 0.05), higher education (*P <* 0.05), more senior professional title (*P <* 0.01), higher nursing grade (p < 0.05), prior training on IC (*P <* 0.001), participation in geriatric care training (*P <* 0.001), working in hospitals that support new technologies in geriatric nursing (*P <* 0.001), and willingness to receive future training (*P <* 0.001). The most strongly endorsed knowledge items were Z18 (41.9% “strongly agree”; early recognition and intervention of IC decline can improve patients’ quality of life) and Z20 (39.4% “strongly agree”; early promotion of nutrition, exercise, and chronic disease management can reduce IC decline). In contrast, the least understood items were Z5 (26.6% “uncertain”; how to diagnose IC impairment), Z3 (26.4% “uncertain”; how to evaluate IC), and Z7 (25.1% “uncertain”; knowledge of IC assessment tools and their advantages/disadvantages) (Table 2).

**Table 2:** Answers to knowledge dimension items.

Answers to knowledge dimension items(n = 606)						
Topic	Score situation	Very agree	Agree	Indeterminacy	Against	Very against
Z1	2.01 ± 0.786	165(27.2%)	289(47.7%)	138(22.8%)	11(1.8%)	3(0.5%)
Z2	2.03 ± 0.792	158(26.1%)	288(47.5%)	146(24.1%)	10(1.7%)	4(0.7%)
Z3	2.08 ± 0.785	145(23.9%)	287(47.4%)	160(26.4%)	11(1.8%)	3(0.5%)
Z4	2.02 ± 0.765	150(24.8%)	309(51.0%)	133(21.9%)	11(1.8%)	3(0.5%)
Z5	2.08 ± 0.798	149(24.6%)	280(46.2%)	161(26.6%)	13(2.1%)	3(0.5%)
Z6	2.04 ± 0.779	151(24.9%)	295(48.7%)	146(24.1%)	11(1.8%)	3(0.5%)
Z7	2.06 ± 0.802	153(25.2%)	283(46.7%)	152(25.1%)	15(2.5%)	3(0.5%)
Z8	1.87 ± 0.708	186(30.7%)	323(53.3%)	90(14.9%)	5(0.8%)	2(0.3%)
Z9	1.93 ± 0.731	171(28.2%)	317(52.3%)	109(18.0%)	7(1.2%)	2(0.3%)
Z10	1.95 ± 0.741	168(27.7%)	315(52.0%)	112(18.5%)	9(1.5%)	2(0.3%)
Z11	1.90 ± 0.709	171(28.2%)	336(55.4%)	89(14.7%)	8(1.3%)	2(0.3%)
Z12	1.88 ± 0.697	175(28.9%)	335(55.3%)	89(14.7%)	5(0.8%)	2(0.3%)
Z13	1.87 ± 0.680	177(29.2%)	335(55.3%)	91(15.0%)	1(0.2%)	2(0.3%)
Z14	1.89 ± 0.683	169(27.9%)	339(55.9%)	94(15.5%)	2(0.3%)	2(0.3%)
Z15	1.89 ± 0.708	173(28.5%)	334(55.1%)	93(15.3%)	2(0.3%)	4(0.7%)
Z16	1.90 ± 0.692	171(28.2%)	332(54.8%)	99(16.3%)	2(0.3%)	2(0.3%)
Z18	1.65 ± 0.607	254(41.9%)	315(52.0%)	36(5.9%)	0(0.0%)	1(0.2%)
Z20	1.70 ± 0.652	239(39.4%)	316(52.1%)	47(7.8%)	2(0.3%)	2(0.3%)

The mean attitude score was 20.86 ± 5.49 (range: 11–35). Higher scores were observed among nurses who had received IC training (*P <* 0.001), participated in geriatric care training (*P <* 0.001), worked in hospitals supporting new geriatric nursing technologies (*P <* 0.001), perceived their current knowledge as meeting clinical needs (*P <* 0.001), and expected further training (*P <* 0.001). The most strongly endorsed items were T4 (34.0% “agree/strongly agree”; nurses should dynamically monitor older patients’ IC) and T10 (31.5% “agree/strongly agree”; nurses should educate patients and families about IC) (Table 3).

**Table 3:** Answers to attitude dimension items.

Answers to attitude dimension items(n = 606)						
Topic	Score situation	Very agree	Agree	Indeterminacy	Against	Very against
T1	1.80 ± 0.656	200(33.0%)	333(55.0%)	69(11.4%)	4(0.7%)	0(0.0%)
T2	1.80 ± 0.636	196(32.3%)	339(55.9%)	70(11.6%)	1(0.2%)	0(0.0%)
T3	1.76 ± 0.641	211(34.8%)	334(55.1%)	57(9.4%)	4(0.7%)	0(0.0%)
T4	1.75 ± 0.616	206(34.0%)	349(57.6%)	49(8.1%)	1(0.2%)	1(0.2%)
T5	1.74 ± 0.616	210(34.7%)	344(56.8%)	51(8.4%)	0(0.0%)	1(0.2%)
T6	2.88 ± 1.300	58(9.6%)	261(43.1%)	102(16.8%)	67(11.1%)	118(19.5%)
T7	1.89 ± 0.687	175(28.9%)	331(54.6%)	94(15.5%)	6(1.0%)	0(0.0%)
T8	1.82 ± 0.642	188(31.0%)	345(56.9%)	70(11.6%)	3(0.5%)	0(0.0%)
T9	1.81 ± 0.628	187(30.9%)	351(57.9%)	66(10.9%)	2(0.3%)	0(0.0%)
T10	1.78 ± 0.599	191(31.5%)	359(59.2%)	56(9.2%)	0(0.0%)	0(0.0%)
T11	1.85 ± 0.699	190(31.4%)	326(53.8%)	82(13.5%)	7(1.2%)	1(0.2%)

The mean practice score was 32.69 ± 8.70 (range: 16–72). Higher practice scores were observed among nurses with specialist certificates (*P <* 0.05), those who had received IC training (*P <* 0.001), participated in geriatric care training (*P <* 0.001), worked in supportive hospitals *P <* 0.001), had higher proportions of elderly patients in their caseload (*P <* 0.05), perceived their knowledge as clinically sufficient (p < 0.001), and expected future training (*P <* 0.05). However, 28.8% reported that they would not conduct further multidisciplinary evaluations for patients with decreased IC, and 25.2% were unsure about the necessity of evaluating IC in clinical practice (Table 4).

**Table 4:** Answers to practice dimension items.

Answers to practice dimension items(n = 606)						
Topic	Score situation	Always	Occasionally	Generally	Rarely	Never
X1	1.97 ± 0.757	169(27.9%)	302(49.8%)	124(20.5%)	9(1.5%)	2(0.3%)
X2	2.32 ± 0.973	83(13.7%)	349(57.6%)	110(18.2%)	27(4.5%)	37(6.1%)
X3	2.11 ± 0.844	145(23.9%)	283(46.7%)	153(25.2%)	17(2.8%)	8(1.3%)
X4	1.99 ± 0.769	157(25.9%)	316(52.1%)	117(19.3%)	12(2.0%)	4(0.7%)
X5	1.92 ± 0.719	165(27.2%)	342(56.4%)	88(14.5%)	7(1.2%)	4(0.7%)
X6	1.89 ± 0.700	173(28.5%)	339(55.9%)	85(14.0%)	7(1.2%)	2(0.3%)
X7	1.89 ± 0.693	169(27.9%)	347(57.3%)	82(13.5%)	5(0.8%)	3(0.5%)
X8	1.93 ± 0.721	163(26.9%)	333(55.0%)	102(16.8%)	4(0.7%)	4(0.7%)
X9	1.92 ± 0.714	163(26.9%)	341(56.3%)	93(15.3%)	5(0.8%)	4(0.7%)
X10	1.92 ± 0.700	159(26.2%)	347(57.3%)	93(15.3%)	3(0.5%)	4(0.7%)
X11	1.98 ± 0.730	150(24.8%)	332(54.8%)	116(19.1%)	3(0.5%)	5(0.8%)
X12	1.97 ± 0.737	159(26.2%)	319(52.6%)	119(19.6%)	6(1.0%)	3(0.5%)
X13	2.36 ± 0.957	77(12.7%)	327(54.0%)	142(23.4%)	25(4.1%)	35(5.8%)
X14	2.26 ± 0.899	80(13.2%)	364(60.1%)	114(18.8%)	19(3.1%)	29(4.8%)
X15	2.27 ± 0.904	80(13.2%)	364(60.1%)	112(18.5%)	21(3.5%)	29(4.8%)
X16	2.00 ± 0.755	147(24.3%)	329(54.3%)	119(19.6%)	4(0.7%)	7(1.2%)

Univariate analysis indicated significant differences in knowledge scores by age, years of service, education, professional title, nursing level, prior IC training, participation in geriatric care training, hospital support for geriatric nursing innovation, perceived adequacy of knowledge, and expectation of training (all *P <* 0.05). Nurses aged 36–45 had the highest knowledge scores, while those under 25 had the lowest (*P <* 0.01). Nurses with more senior professional titles also scored higher (*P <* 0.01). Interestingly, nurses who reported being “not satisfied” or “completely dissatisfied” with their current knowledge needs had the highest knowledge scores. This seemingly paradoxical result may reflect that nurses with greater knowledge are more aware of the complexity of IC and thus more critical of their own preparedness.

For attitudes, significant group differences were observed (*P <* 0.001) across prior IC training, participation in geriatric care training, hospital support, adequacy of knowledge, and expectation of training. Similar to knowledge, nurses who expressed dissatisfaction with their knowledge needs had higher attitude scores, which may suggest that increased awareness drives stronger recognition of the importance of IC.

For practices, significant differences were observed by the proportion of elderly patients cared for in the past year (*P <* 0.05). Nurses with 0–25% elderly patients had the highest practice scores, while those with >75% had the lowest (*P <* 0.05). This may reflect the higher workload and burnout experienced when caring for a predominantly elderly population.

Multicollinearity tests confirmed acceptable tolerance values (>0.1) and VIF values (<2).

**Fig 1:**
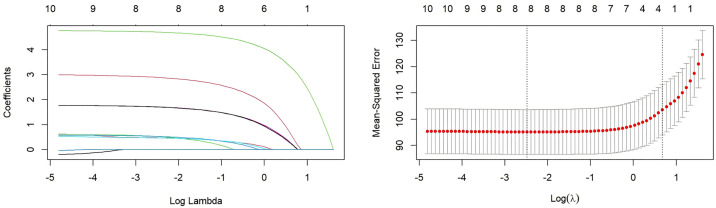
LASSO regression for variable selection with the knowledge score as the dependent variable. **(A)** The coefficient distribution plot shows the trajectory of each variable coefficient as the penalty parameter (log(λ)) increases. Each curve represents a variable. The vertical dashed line indicates the value of lambda selected via 10-fold cross-validation (lambda.min). **(B)** The cross-validation curve plot shows the mean-squared error (MSE) across different values of log(λ). The left vertical dashed line (lambda.min) indicates the λ value that gives the minimum MSE. The right vertical dashed line (lambda.1se) indicates the largest value of λ such that the error is within one standard error of the minimum. The numbers at the top of the plot indicate the number of non-zero coefficients at that point. As shown in the figure: seniority(1), education(2), nursing level(3), Received training on internal capacity(4), Participated in geriatric nursing related training(5), hospital support for geriatric nursing innovation(6), perceived adequacy of knowledge(7), and expected frequency of receiving training(8).

**Fig 2:**
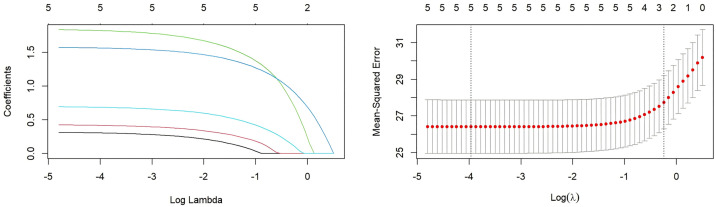
LASSO regression for variable selection with the attitude score as the dependent variable. **(A)** The coefficient distribution plot shows the trajectory of each variable coefficient as the penalty parameter (log(λ)) increases. Each curve represents a variable. The vertical dashed line indicates the value of lambda selected via 10-fold cross-validation (lambda.min). **(B)** The cross-validation curve plot shows the mean-squared error (MSE) across different values of log(λ). The left vertical dashed line (lambda.min) indicates the λ value that gives the minimum MSE. The right vertical dashed line (lambda.1se) indicates the largest value of λ such that the error is within one standard error of the minimum. The numbers at the top of the plot indicate the number of non-zero coefficients at that point. As shown in the figure: received training(1), Participated in geriatric nursing related training(2), hospital support(3), perceived adequacy of knowledge(4), and expectation of training(5).

**Fig 3:**
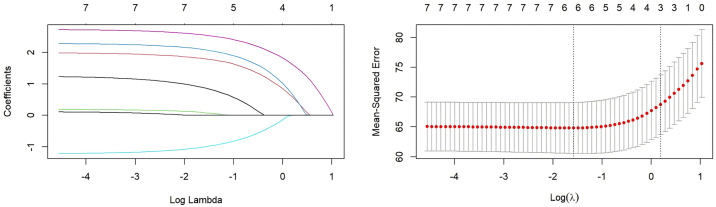
LASSO regression for variable selection with the practice score as the dependent variable. **(A)** The coefficient distribution plot shows the trajectory of each variable coefficient as the penalty parameter (log(λ)) increases. Each curve represents a variable. The vertical dashed line indicates the value of lambda selected via 10-fold cross-validation (lambda.min). **(B)** The cross-validation curve plot shows the mean-squared error (MSE) across different values of log(λ). The left vertical dashed line (lambda.min) indicates the λ value that gives the minimum MSE. The right vertical dashed line (lambda.1se) indicates the largest value of λ such that the error is within one standard error of the minimum. The numbers at the top of the plot indicate the number of non-zero coefficients at that point. As shown in the figure: have a specialized certificate(1), received training(2), Participated in geriatric nursing related training(3), hospital support(4), Proportion of patients aged 60 and above(5), perceived adequacy of knowledge(6), and expectation of training(7).

LASSO regression was performed to identify independent predictors of knowledge, attitude, and practice scores. The results indicated that: (1) Knowledge was associated with seniority, educational attainment, nursing hierarchy, receipt of training on intrinsic capacity, participation in geriatric nursing–related training, perceived hospital support for geriatric nursing innovation, perceived adequacy of existing knowledge, and expected frequency of training (Fig 1). (2) Attitude was associated with receipt of training, participation in geriatric nursing–related training, hospital support, perceived adequacy of existing knowledge, and expectation of training (Fig 2). (3) Practice was associated with possession of a specialized certificate, receipt of training, participation in geriatric nursing–related training, hospital support, the proportion of patients aged ≥60 cared for in the past year, perceived adequacy of knowledge, and expectation of training (Fig 3).

Correlation analysis revealed strong positive associations between knowledge and attitude (*r* = 0.732, *P <* 0.01), knowledge and practice (*r* = 0.617,*P <* 0.01), and attitude and practice (*r* = 0.666, *P <* 0.01) (Table 5). The bootstrap mediation effect test results indicated an indirect effect (*B* = 0.421, *95%CI*:0.296–0.558) and a direct effect (*B* = 0.295, *95%CI:* 0.146–0.451), with effect proportions of 59% and 41%, respectively (Table 6). The fit indices of the structural equation model constructed using AMOS indicated that all adjusted goodness-of-fit metrics of the revised model met the established evaluation criteria (Table 7).

**Table 5:** Correlations among knowledge, attitude, and practice.

Correlations			
	Practice	Knowledge	Attitude
Practice	1		
Knowledge	.617**	1	
Attitude	.666**	.732**	1

** Correlation is significant at the 0.01 level(2-tailed).

**Table 6:** Bootstarp Mediation effect test results.

Parameter	Estimate	95%CI	*P*	Proportion of Effect
indirect effect	0.421	0.296 ~ 0.558	0.000	59%
direct effect	0.295	0.146 ~ 0.451	0.000	41%
Total effect	0.715	0.592 ~ 0.846	0.000	

**Table 7:** Goodness-of-fit test results of the modified model.

Goodness-of-Fit Test Results of the Modified Model			
Statistical measures	Goodness-of-fit criteria	results	Model fit assessment
PCMIN/DF	1 ~ 5	4.171	Yes
RMSEA	<0.08	0.072	Yes
TLI	>0.90	0.908	Yes
IFI	>0.90	0.916	Yes
CFI	>0.90	0.915	Yes

## 5. Discussion

This study provides novel insights into the knowledge, attitudes, and practices (KAP) of clinical nurses regarding Intrinsic Capacity (IC) in older adults in Xinjiang. The findings reveal generally low knowledge, but moderate attitudes and practices, with substantial variability across subgroups, underscoring both challenges and opportunities for strengthening geriatric nursing within the IC framework.

The limited knowledge observed among nurses, particularly regarding IC assessment, diagnostic methods, and intervention strategies, reflects a broader gap in professional education and clinical training. Many participants expressed uncertainty about evaluation tools and procedures (Z3, Z5, Z7), highlighting insufficient integration of IC into current nursing curricula and hospital-based training. This is concerning given that IC decline has been identified as a key predictor of adverse health outcomes, including functional decline, falls, and mortality in older adults [5,18,21,24–27,35–37]. Enhancing nurses’ conceptual and practical understanding of IC should therefore be a priority for clinical education and policy development.

An interesting and seemingly paradoxical finding is that nurses who reported being “not satisfied” or “completely dissatisfied” with their IC-related knowledge needs achieved higher knowledge scores. Rather than indicating a contradiction, this may suggest that more knowledgeable nurses are also more aware of the complexity of IC and more critical of their own preparedness, consistent with existing educational theory. This heightened awareness underscores the need for structured guidelines and practical frameworks to transform theoretical knowledge into clinical confidence and competence [22].

Attitudes and practices were positively associated with prior IC-related training, participation in geriatric care programs, and employment in hospitals supporting geriatric nursing innovation. These findings emphasize the importance of institutional support and professional development opportunities in translating knowledge into attitudes and practice behaviors [12,30,38,39]. Hospitals promoting geriatric innovation provide an environment in which IC assessment and intervention can be normalized and integrated into routine care.

Demographic differences further refine the interpretation of these results. Nurses under 35 years of age had lower knowledge scores, likely reflecting limited clinical exposure and fewer training opportunities. Conversely, nurses with higher professional titles and longer work experience demonstrated higher knowledge and more positive attitudes, suggesting cumulative professional experience as a protective factor. Tailored interventions, such as early-career training, mentorship, and structured continuing education, may help address these gaps and ensure that junior nurses are equipped with the competencies necessary for IC-oriented care [13,40,41].

Practice scores were inversely related to the proportion of elderly patients in the nurses’ caseload, potentially reflecting occupational strain, time constraints, and emotional fatigue. These findings highlight the need for workload management and supportive organizational structures to enable nurses to implement IC-related practices effectively, particularly in high-demand settings [31].

Structural equation modeling confirmed positive relationships among KAP dimensions: knowledge positively influenced both attitude and practice, and attitude positively influenced practice. This model reinforces that improving knowledge can generate broader benefits by enhancing both perceptions and behaviors. Nevertheless, knowledge alone is insufficient if not supported by institutional resources, standardized tools, and continuous reinforcement mechanisms [42–45].

Overall, these findings suggest that effective strategies to improve nurses’ engagement with IC should include: (1) systematic, targeted training emphasizing IC concepts, assessment methods, and interventions; (2) institutional support through policy initiatives and departmental promotion of geriatric innovation; (3) tailored interventions for younger and less experienced nurses; and (4) organizational measures to manage workload and prevent burnout. By addressing these areas, clinical nursing can better support early recognition and management of IC decline in older patients, ultimately contributing to healthy aging [5,18,21–27,31,35–41].

## 6. Conclusion

In summary, this study indicates that the participating nurses demonstrated relatively limited knowledge, and only moderate attitudes and practices, regarding the Intrinsic Capacity (IC) of older adults. The findings suggest that awareness of IC, particularly in terms of early symptom recognition, assessment approaches, and intervention strategies, remains insufficient among this sample of clinical nurses. This highlights the potential need for tailored training and educational initiatives to strengthen their competence in this area. In addition, some nurses reported less positive attitudes or even tendencies toward neglect, which may be related to work-related stress, emotional fatigue, or uncertainty about effective care strategies. Interventions such as professional workshops, seminars, and case-based learning could help improve nurses’ knowledge and confidence in supporting the maintenance of IC in older patients.

Given the rapid aging of the population, maintaining intrinsic capacity has become an increasingly important challenge for clinical nursing practice. As frontline caregivers, nurses’ knowledge, attitudes, and practices are critical for optimizing patient care and supporting healthy aging. Nevertheless, it is important to acknowledge that this study was based on a cross-sectional survey within a specific context, and the results should not be generalized to national trends without further multicenter and longitudinal research. Future studies with larger and more diverse samples are warranted to validate and extend these findings.

**Fig 4:**
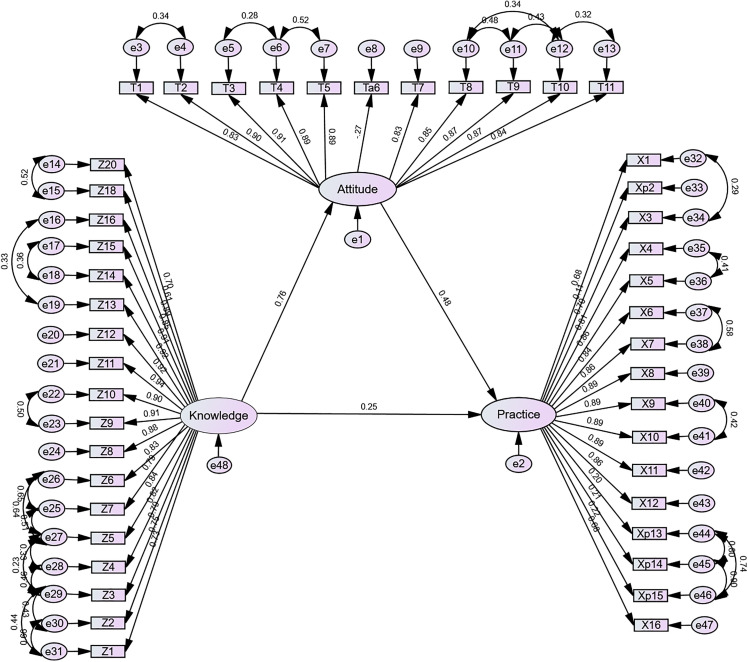
The structural equation model of factors influencing clinical nurses’ KAP regarding intrinsic capacity. Fig 4 presents the structural equation model illustrating the relationships among demographic characteristics, work experience, training, and the knowledge, attitude, and practice (KAP) of clinical nurses toward intrinsic capacity in older adults. In the model, rectangles represent observed variables and ovals represent latent constructs. Single-headed arrows denote hypothesized directional paths, with standardized path coefficients shown alongside. Double-headed arrows indicate correlations. The model fit indices were as follows: χ²/df = 4.171, CFI = 0.915, TLI = 0.908, and RMSEA = 0.072, all of which indicate an acceptable model fit. Abbreviations: IC, Intrinsic Capacity; KAP, Knowledge, Attitude, and Practice.

The structural equation modeling framework presented in Fig 4 provides an initial exploration of the hypothesized relationships among KAP constructs. While the path directions and regression weights offer insight into potential associations, further research is required to confirm these relationships in broader clinical settings.

## Supporting information

S1 File Questionnaire—Chinese. (DOCX)

S2 File Questionnaire—English. (DOCX)

S3 File All minimal dataset. (ZIP)
