# Supplementary material for: Clinical nurse’s knowledge, attitude, and practice regarding the Intrinsic Capacity of the aged: A cross-sectional study
Source: PLoS One. 2026 Mar 19;21(3):e0330471. doi: 10.1371/journal.pone.0330471 (PMC13001941; doi:10.1371/journal.pone.0330471)
Supplement: S1 File — (DOCX) [file pone.0330471.s001.docx]

**临床护士对老年患者内在能力知信行问卷**

尊敬的护士同仁：

        您好，感谢您参与本次调查！本研究旨在调查护理人员对老年患者内在能力知识、态度、行为现状及主要影响因素，以增进护理人员对老年患者内在能力及相关知识的关注，从而改善护理人员对内在能力下降的老年患者的态度及行为，从而为缓解甚至逆转内在能力下降制定针对性的培训提供依据。本次调查匿名，所有数据仅用于调查，遵循保密原则，不会泄露个人信息，请您如实独立填写，衷心感谢您对本研究的支持！如果您同意参与本研究，请继续答题，即默认为您知情同意，反之您可选择直接退出答题系统，谢谢！

第一部分

一般调查资料

1.您的性别 [单选题] *

| ○男 |
| --- |
| ○女 |

2.您的年龄是( )岁 [填空题] *

_________________________________

3.您的族别 [单选题] *

| ○汉族 |
| --- |
| ○回族 |
| ○维吾尔族 |
| ○其它: _________________ |

4.您的工龄是( )年 [填空题] *

_________________________________

5.您的最高学历 [单选题] *

| ○中专 |
| --- |
| ○大专 |
| ○本科 |
| ○研究生及以上 |

6.您的职称 [单选题] *

| ○护士 |
| --- |
| ○护理师 |
| ○主管护师 |
| ○副主任护师 |
| ○主任护师 |

7.您的工作能级: [单选题] *

| ○护士，未分等级 |
| --- |
| ○N0 |
| ○N1 |
| ○N2 |
| ○N3 |
| ○N4及以上 |

8.您的职务 [单选题] *

| ○无职务 |
| --- |
| ○总带教 |
| ○护理组组长 |
| ○护士长 |
| ○其他: _________________ |

9.您是否取得专科护士证书? [单选题] *

| ○是 |
| --- |
| ○否 |

10.您取得专科护士证书的类型? [单选题] *

| ○未取得 |
| --- |
| ○急诊急救专科护士 |
| ○重症专科护士 |
| ○呼吸治疗师 |
| ○其他: _________________ |

11.您取得专科护士证书的级别? [单选题] *

| ○院内证书 |
| --- |
| ○自治区级证书 |
| ○国家级证书 |
| ○其他: _________________ |

12.您家中有≥60岁的父亲/母亲 [单选题] *

| ○是 |
| --- |
| ○否 |

13.您所在科室名称: [填空题] *

_________________________________

14.您所在单位名称: [填空题] *

_________________________________

15.您所在医院级别 [单选题] *

| ○二级医院 |
| --- |
| ○三级医院 |
| ○其它: _________________ |

16.您是否接受过老年人内在能力管理相关知识的培训: [单选题] *

| ○是 |
| --- |
| ○否 |

17.您是否参与过老年诊疗/护理相关学习班、进修培训或专科培训项目? [单选题] *

| ○是 |
| --- |
| ○否 |

18.医院是否对您开展老年医疗/护理相关工作给予积极的支持（包括但不限于政策鼓励、外出学习等）? [单选题] *

| ○是 |
| --- |
| ○否 |

19.您在工作期间照护≥60岁老年患者比例大约占多少? [单选题] *

| ○0~25% |
| --- |
| ○26~50% |
| ○51~75% |
| ○76%~100% |

20.以下哪些是识别内在能力下降的实际障碍（根据您在工作中面临的障碍回答，而不是潜在的和理论性的障碍） [多选题] *

| □缺乏时间 |
| --- |
| □对我所照护的对象缺乏全面的了解（全面的病史，对社会环境的了解） |
| □缺乏内在能力领域的专业知识 |
| □缺乏临床技能和对内在能力的了解 |
| □缺少信息资源 |
| □内在能力筛查是无用的，因为没有治愈的方法 |
| □内在能力筛查是无用的，因为即便筛查到什么，我不知晓如何干预 |
| □老年人不感兴趣/不同意评估其内在能力状态 |
| □老年人患有其他我认为更重要的病理状况 |
| □其它（请具体写出）： _________________ |

21.实际上在应用管理内在能力的过程中所面临的障碍是什么(根据您在中作中遇到的障碍回答，而不是潜在和理论性的障碍) [多选题] *

| □缺乏时间 |
| --- |
| □对我所照护的对象缺乏全面的了解(全面的病史，对社会环境的了解) |
| □缺乏内在能力领域的专业知识 |
| □缺乏临床技能和对内在能力的了解 |
| □缺乏内在能力有效的干预方法 |
| □缺乏有效的跨专业合作 |
| □医疗场所中缺乏使用内在能力管理技术的基础设施 |
| □缺乏支持性的人文环境 |
| □资金支持不足 |
| □老年人缺乏动力或依从性不足 |
| □其它（请具体写出）： _________________ |

22.您认为目前掌握的内在能力知识是否满足临床工作 [单选题] *

| ○完全满足 |
| --- |
| ○大部分满足 |
| ○基本满足 |
| ○不太满足 |
| ○完全不满足 |

23.您期望接受的内在能力培训内容 [多选题] *

| □内在能力筛查与评估 |
| --- |
| □内在能力的概念及进展轨迹 |
| □内在能力的干预措施 |
| □内在能力的危险因素 |
| □内在能力研究的最新进展 |
| □其他（请具体写出）： _________________ |

24.您期望的内在能力培训频率 [单选题] *

| ○每个月 |
| --- |
| ○每季度 |
| ○每半年 |
| ○每年 |

25.您期望的内在能力培训方式 [多选题] *

| □下发文件自学 |
| --- |
| □学术会议、讲座 |
| □短期培训班 |
| □科室业务学习 |
| □专业书籍和文献 |
| □互联网在线学习 |
| □其他（请具体写出）： _________________ |

第二部分

一、知识维度（主观部分）

1.我知晓老年人内在能力的概念。 [单选题] *

| ○非常同意 |
| --- |
| ○同意 |
| ○不确定 |
| ○反对 |
| ○非常反对 |

2.我知晓老年人内在能力包含的不同维度。 [单选题] *

| ○非常同意 |
| --- |
| ○同意 |
| ○不确定 |
| ○反对 |
| ○非常反对 |

3.我知晓如何评估老年人内在能力。 [单选题] *

| ○非常同意 |
| --- |
| ○同意 |
| ○不确定 |
| ○反对 |
| ○非常反对 |

4.我知晓内在能力的危险因素有哪些。 [单选题] *

| ○非常同意 |
| --- |
| ○同意 |
| ○不确定 |
| ○反对 |
| ○非常反对 |

5.我知晓如何诊断内在能力受损。 [单选题] *

| ○非常同意 |
| --- |
| ○同意 |
| ○不确定 |
| ○反对 |
| ○非常反对 |

6.我知晓内在能力的预防措施有哪些。 [单选题] *

| ○非常同意 |
| --- |
| ○同意 |
| ○不确定 |
| ○反对 |
| ○非常反对 |

7.我知晓内在能力的评估工具有哪些,并清楚不同评估工具的优缺点。 [单选题] *

| ○非常同意 |
| --- |
| ○同意 |
| ○不确定 |
| ○反对 |
| ○非常反对 |

8.我知晓内在能力下降与日常生活能力下降、躯体活动受限、跌倒、入院风险及死亡等不良健康结局密切相关 [单选题] *

| ○非常同意 |
| --- |
| ○同意 |
| ○不确定 |
| ○反对 |
| ○非常反对 |

9.我知晓内在能力作为一种动态结构，反映了整个生命周期中功能轨迹的改变。可分为3个连续阶段，包含内在能力水平高且稳定、功能衰退以及严重失能且照护依赖阶段。 [单选题] *

| ○非常同意 |
| --- |
| ○同意 |
| ○不确定 |
| ○反对 |
| ○非常反对 |

10. 我知晓内在能力的多维度、异质性及其与一个人生活的不同方面(包括共病、功能能力、身体健康、心理社会健康和认知功能)存在复杂的多向关系 [单选题] *

| ○非常同意 |
| --- |
| ○同意 |
| ○不确定 |
| ○反对 |
| ○非常反对 |

11.我知晓内在能力的轨迹受生活方式等因素的影响，认知障碍、跌倒、活动障碍以及多重用药与内在能力之间存在复杂多向关系 [单选题] *

| ○非常同意 |
| --- |
| ○同意 |
| ○不确定 |
| ○反对 |
| ○非常反对 |

12.我知晓老年患者的疾病和管理、运动干预和营养干预，认知干预、心理社会支持及多领域综合干预对内在能力的管理也十分重要 [单选题] *

| ○非常同意 |
| --- |
| ○同意 |
| ○不确定 |
| ○反对 |
| ○非常反对 |

13.我知晓老年患者内在能力的维持和逆转需要进一步进行老年综合评估并寻求多学科合作干预。 [单选题] *

| ○非常同意 |
| --- |
| ○同意 |
| ○不确定 |
| ○反对 |
| ○非常反对 |

14.内在能力是老年患者不可避免的结果，随年龄的增长呈现不断下降的趋势。 [单选题] *

| ○非常同意 |
| --- |
| ○同意 |
| ○不确定 |
| ○反对 |
| ○非常反对 |

15.内在能力会增加再患病率、非计划再住院率，增加患者的病痛，降低患者的生活质量。 [单选题] *

| ○非常同意 |
| --- |
| ○同意 |
| ○不确定 |
| ○反对 |
| ○非常反对 |

16.内在能力是老年人功能发挥的基础，是指个体所有的生理能力和心理能力的总和。 [单选题] *

| ○非常同意 |
| --- |
| ○同意 |
| ○不确定 |
| ○反对 |
| ○非常反对 |

17.内在能力主要包括哪几个维度。 [多选题] *

| □认知 |
| --- |
| □心理 |
| □听力 |
| □视力 |
| □活力 |
| □运动 |
| □不清楚 |

18.早期识别、干预、预防内在能力进展，可提高老年患者的生活质量，对老年患者、家庭和社会产生很大益处。 [单选题] *

| ○非常同意 |
| --- |
| ○同意 |
| ○不确定 |
| ○反对 |
| ○非常反对 |

19.内在能力的下降与以下哪些因素有关。 [多选题] *

| □个人血型 |
| --- |
| □工作类型 |
| □年龄 |
| □性别 |
| □婚姻状况 |
| □文化程度 |
| □健康行为 |
| □共病数量 |
| □社会支持 |
| □居住方式 |
| □营养不良 |
| □不确定 |

20.早期促使老年患者合理的营养、运动以及慢性病的管理是预防或减少老年患者内在能力有效的干预措施。 [单选题] *

| ○非常同意 |
| --- |
| ○同意 |
| ○不确定 |
| ○反对 |
| ○非常反对 |

第二部分

二、态度维度

1.您认为医护人员如果对内在能力引起足够的重视可逆转或延缓内在能力的下降 [单选题] *

| ○非常同意 |
| --- |
| ○同意 |
| ○不确定 |
| ○反对 |
| ○非常反对 |

2.您认为改善老年患者内在能力的状态需要医护人员采取行动 [单选题] *

| ○非常同意 |
| --- |
| ○同意 |
| ○不确定 |
| ○反对 |
| ○非常反对 |

3.您认为医护人员维持、阻止甚至逆转内在能力的下降对老年患者疾病控制、生活质量的提高非常重要。 [单选题] *

| ○非常重要 |
| --- |
| ○重要 |
| ○不确定 |
| ○不重要 |
| ○非常不重要 |

4.您认为医护人员应动态观察老年患者的内在能力状况。 [单选题] *

| ○非常同意 |
| --- |
| ○同意 |
| ○不确定 |
| ○反对 |
| ○非常反对 |

5.您认为医护人员应该接受正规的老年内在能力知识培训。 [单选题] *

| ○非常同意 |
| --- |
| ○同意 |
| ○不确定 |
| ○反对 |
| ○非常反对 |

6.您认为医护人员应该对老年内在能力下降患者开展多领域的干预项目(尤其是运动与营养相结合) [单选题] *

| ○非常不同意 |
| --- |
| ○不同意 |
| ○不确定 |
| ○同意 |
| ○非常同意 |

7.您认为医护人员应该担负起评估老年患者内在能力的护理工作。 [单选题] *

| ○非常同意 |
| --- |
| ○同意 |
| ○不确定 |
| ○反对 |
| ○非常反对 |

8.您认为早期功能锻炼对老年内在能力的维持及逆转非常重要。 [单选题] *

| ○非常同意 |
| --- |
| ○同意 |
| ○不确定 |
| ○反对 |
| ○非常反对 |

9.您认为医护人员应该像重视其他症状一样（如DVT），重视对老年内在能力下降的预防。 [单选题] *

| ○非常同意 |
| --- |
| ○同意 |
| ○不确定 |
| ○反对 |
| ○非常反对 |

10.您认为在临床工作中需对患者或家属进行老年患者内在能力相关知识的教育。 [单选题] *

| ○非常同意 |
| --- |
| ○同意 |
| ○不确定 |
| ○反对 |
| ○非常反对 |

11.您认为老年患者的内在能力状况应纳入临床工作中的交接班内容。 [单选题] *

| ○非常同意 |
| --- |
| ○同意 |
| ○不确定 |
| ○反对 |
| ○非常反对 |

第二部分

三、行为维度

1.您是否在临床工作中会主动关注老年患者的内在能力状况? [单选题] *

| ○总是 |
| --- |
| ○是 |
| ○一般 |
| ○不是 |
| ○从未 |

2.您会考虑对老年内在能力下降患者进行进一步的多学科团队协作的综合评估 [单选题] *

| ○非常不同意 |
| --- |
| ○不同意 |
| ○不确定 |
| ○同意 |
| ○非常同意 |

3.您是否在临床工作中对老年患者进行内在能力评估? [单选题] *

| ○总是 |
| --- |
| ○是 |
| ○一般 |
| ○不是 |
| ○从未 |

4.您是否会及时关注和评估老年患者的肌肉肌力状况? [单选题] *

| ○总是 |
| --- |
| ○是 |
| ○一般 |
| ○不是 |
| ○从未 |

5.您是否会及时关注和评估老年患者的认知状况? [单选题] *

| ○总是 |
| --- |
| ○是 |
| ○一般 |
| ○不是 |
| ○从未 |

6.您是否会及时关注和评估老年患者的视力状况? [单选题] *

| ○总是 |
| --- |
| ○是 |
| ○一般 |
| ○不是 |
| ○从未 |

7.您是否会及时关注和评估老年患者的听力状况? [单选题] *

| ○总是 |
| --- |
| ○是 |
| ○一般 |
| ○不是 |
| ○从未 |

8.您是否会及时关注和评估老年患者的心理状况? [单选题] *

| ○总是 |
| --- |
| ○是 |
| ○一般 |
| ○不是 |
| ○从未 |

9.您是否会为老年患者提供有效的早期功能锻炼指导? [单选题] *

| ○总是 |
| --- |
| ○是 |
| ○一般 |
| ○不是 |
| ○从未 |

10.您是否会指导家属帮助患者进行适当活动以缓解躯体乏力等症状? [单选题] *

| ○总是 |
| --- |
| ○是 |
| ○一般 |
| ○不是 |
| ○从未 |

11.您是否对患者早期活动的护理干预后及时进行评价? [单选题] *

| ○总是 |
| --- |
| ○是 |
| ○一般 |
| ○不是 |
| ○从未 |

12.您是否对患者实施营养干预后及时进行评价? [单选题] *

| ○总是 |
| --- |
| ○是 |
| ○一般 |
| ○不是 |
| ○从未 |

13.您会对老年内在能力下降患者进行基于证据干预措施的指导 [单选题] *

| ○非常不同意 |
| --- |
| ○不同意 |
| ○不确定 |
| ○同意 |
| ○非常同意 |

14.您会对内在能力下降的老年患者、家属/主要照护者进行内在能力相关的健康教育或培训 [单选题] *

| ○非常不同意 |
| --- |
| ○不同意 |
| ○不确定 |
| ○同意 |
| ○非常同意 |

15.我会与其它健康服务提供者交流内在能力相关的评估或知识 [单选题] *

| ○非常不同意 |
| --- |
| ○不同意 |
| ○不确定 |
| ○同意 |
| ○非常同意 |

16.您是否在工作过程中进行内在能力相关知识的积累? [单选题] *

| ○总是 |
| --- |
| ○是 |
| ○一般 |
| ○不是 |
| ○从未 |
