# Supplementary material for: Clinical nurse’s knowledge, attitude, and practice regarding the Intrinsic Capacity of the aged: A cross-sectional study
Source: PLoS One. 2026 Mar 19;21(3):e0330471. doi: 10.1371/journal.pone.0330471 (PMC13001941; doi:10.1371/journal.pone.0330471)
Supplement: S2 File — (DOCX) [file pone.0330471.s002.docx]

**Clinical Nurse's Knowledge, Attitude, and Practice**

**Questionnaire on Intrinsic Competence of Elderly Patients**

Dear Fellow Nurses:

Hello, thank you for participating in this survey! The purpose of this study is to investigate the current status of nursing staff's knowledge, attitude, and practice regarding intrinsic competence of elderly patients and the main influencing factors, in order to increase nursing staff's attention to the intrinsic competence of elderly patients and related knowledge, so as to improve nursing staff's attitude and practice towards elderly patients with declining intrinsic competence, and thus to provide the basis for the development of targeted trainings for alleviating or even reversing the decline in intrinsic competence. This survey is anonymous, all data will be used only for the survey, following the principle of confidentiality, without disclosing personal information, please fill out the survey truthfully and independently, and we sincerely thank you for your support of this study! If you agree to participate in this study, please continue to answer the question, that is, the default for your informed consent, otherwise you can choose to directly exit the answering system, thank you!

Part I

General survey information

1. Your gender [Single choice]*

| ○Male |
| --- |
| ○Female |

2. Your age is ( ) years [fill in the blank] *


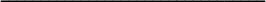


3. Your ethnicity [Single choice]*

| ○ Han ethnic group |
| --- |
| ○ Hui Islamic ethnic group living across China |
| ○ Uighur (Uyghur) ethnic group of Xinjiang |
| ○Other: |

4. Your length of service is ( ) years [fill in the blanks] *


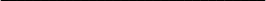


5. Your highest level of education [Single choice]*

| ○Secondary specialized school |
| --- |
| ○Junior college |
| ○Undergraduate |
| ○Graduate and above |

6. Your Professional title [Single choice]*

| ○Nurse |
| --- |
| ○Junior nurse |
| ○Intermediate nurse |
| ○Associate chief nurse |
| ○chief superintendent nurse |

7. Your nursing competence level: [Single choice]*

| ○ Nurse, ungraded |
| --- |
| ○N0 |
| ○N1 |
| ○N2 |
| ○N3 |
| ○N4 and above |

8. Your position [Single choice]*

| ○No position |
| --- |
| ○Total teaching |
| ○ Nursing Team Leader |
| ○head nurse |
| ○Others: |

9. Are you certified as a nurse specialist? [Single choice] *

| ○Yes |
| --- |
| ○No |

10. The type of specialty nurse certification you obtained? [Single choice] *

| ○unachieved |
| --- |
| ○ Nurse Specialist in Emergency and Urgent Care |
| ○Critical Care Nurse Specialist |
| ○respiratory therapist |
| ○Others: |

11.What level of certification did you obtain as a nurse specialist? [Single choice] *

| ○Hospital level |
| --- |
| ○Autonomous Region Certificate |
| ○ National level certificates |
| ○Not obtained: |

12. You have a father/mother ≥ 60 years old in your household [Single choice] *

| ○Yes |
| --- |
| ○No |

13. Name of your department: [fill in the blank] *


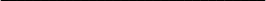


1. Name of your organization: [fill in the blank]

15. Level of your hospital [Single choice]*

| ○ Secondary hospital |
| --- |
| ○Tertiary hospital |
| ○Below level 2: |

16. Have you been trained in the management of the intrinsic capacity of older persons: [Single choice] *

| ○Yes |
| --- |
| ○No |

17. Have you ever participated in a geriatric diagnosis/care related study course, refresher training or specialty training program? [Single choice] *

| ○Yes |
| --- |
| ○No |

18. Does the hospital actively support you in carrying out geriatric medical/nursing related work (including but not limited to policy incentives, study away, etc.)? [Single choice] *

| ○Yes |
| --- |
| ○No |

19.Approximately what percentage of older patients ≥60 years of age do you care for during your workday? [Single choice] *

| ○0~25% |
| --- |
| ○26~50% |
| ○51~75% |
| ○76%~ 100% |

20. Which of the following are actual barriers to recognizing intrinsic diminished capacity (answer based on the barriers you face at work, not potential and theoretical barriers) [Multiple choice] *

| □ lack of time |
| --- |
| □ Lack of comprehensive knowledge of the person in my care  (comprehensive medical history, knowledge of social environment) |
| □ Lack of expertise in areas of intrinsic competence |
| □ Lack of clinical skills and knowledge of intrinsic competencies |
| □ Lack of information resources |
| □Intrinsic ability screening is useless because there is no cure |
| □Screening for intrinsic ability is useless because even if something is screened, I don't know how to intervene |
| □Older adults are not interested in/don't agree with assessing their intrinsic capacity status |
| □ Elderly people with other pathologies that I consider to be more important |
| □Other (please be specific): |

21. What are the obstacles actually faced in applying the inherent competencies of management (answer based on the obstacles you have encountered in your work, not on potential and theoretical obstacles) [Multiple choice] *

| □ lack of time |
| --- |
| □ Lack of comprehensive knowledge of the person in my care  (comprehensive medical history, knowledge of social environment) |
| □ Lack of expertise in areas of intrinsic competence |
| □ Lack of clinical skills and knowledge of intrinsic competencies |
| □ Lack of intrinsic capacity to intervene effectively |
| □ Lack of effective inter-professional cooperation |
| □ Lack of infrastructure for the use of intrinsic capacity management techniques in health-care settings |
| □ Lack of a supportive human environment |
| □Insufficient financial support |
| □ Lack of motivation or adherence in older adults |
| □Other (please be specific): |

22. Do you think that your current knowledge of intrinsic competence is sufficient for clinical work [Single choice] *

| ○ Completely satisfied |
| --- |
| ○Mostly satisfied |
| ○Basically satisfied |
| ○ Not satisfied |
| ○Not satisfied at all |

23. What do you expect to receive in terms of training in intrinsic competence [multiple choice] *

| □Intrinsic capacity screening and assessment |
| --- |
| □Concept of endogenous capacity and trajectory of progress |
| □Interventions for endogenous capacities |
| □ Risk factors for intrinsic capacity |
| □ Recent advances in the study of intrinsic capacity |
| □Other (please be specific): |

24. Your desired frequency of training in intrinsic competencies [Single choice]*

| ○each month |
| --- |
| ○quarterly |
| ○every half year |
| ○annually |

25. Your preferred mode of training in intrinsic competencies [multiple choice] *

| □Issuance of documents for self-study |
| --- |
| □Academic conferences, lectures |
| □Short-term training courses |
| □Section business learning |
| □Specialized books and literature |
| □Internet e-learning |
| □Other (please be specific): |

Part II

I. Knowledge dimension (subjective component)

1. I am aware of the concept of intrinsic capacity of older persons. [Single choice] *

| ○Very agree |
| --- |
| ○Agree |
| ○ Indeterminacy |
| ○Against |
| ○Very against |

2. I am aware of the different dimensions of intrinsic competence of older persons. [Single choice] *

| ○Very agree |
| --- |
| ○Agree |
| ○ Indeterminacy |
| ○Against |
| ○Very against |

| ○Very agree |
| --- |
| ○Agree |
| ○ Indeterminacy |
| ○Against |
| ○Very against |

3. I know how to assess the intrinsic capacity of older people. [Single choice] *

4. I am aware of the risk factors for intrinsic competence. [Single choice] *

| ○Very agree |
| --- |
| ○Agree |
| ○ Indeterminacy |
| ○Against |
| ○Very against |

5. I know how to diagnose impaired intrinsic capacity. [Single choice]

| ○Very agree |
| --- |
| ○Agree |
| ○ Indeterminacy |
| ○Against |
| ○Very against |

| ○Very agree |
| --- |
| ○Agree |
| ○ Indeterminacy |
| ○Against |
| ○Very against |

6. I know what are the precautions for intrinsic capacity. [Single choice]

7. I am aware of the assessment tools for intrinsic competence and the advantages and disadvantages of different assessment tools. [Single choice] *

| ○Very agree |
| --- |
| ○Agree |
| ○ Indeterminacy |
| ○Against |
| ○Very against |

8. I know that reduced intrinsic capacity is strongly associated with adverse health outcomes such as reduced ability to perform activities of daily living, limitation of physical activity, falls, risk of hospitalization and death [Single choice] *

| ○Very agree |
| --- |
| ○Agree |
| ○ Indeterminacy |
| ○Against |
| ○Very against |

9. I am aware that intrinsic capacity, as a dynamic construct, reflects changes in the trajectory of functioning across the life cycle. It can be categorized into 3 successive stages, encompassing high and stable levels of intrinsic capacity, functional decline, and severe disability and care dependency. [Single choice] *

| ○Very agree |
| --- |
| ○Agree |
| ○ Indeterminacy |
| ○Against |
| ○Very against |

10. I am aware of the multidimensionality and heterogeneity of intrinsic capacity and its complex and multidirectional relationship with different aspects of a person's life, including co-morbidities, functional capacity, physical health, psychosocial health and cognitive functioning [Single choice]*

| ○Very agree |
| --- |
| ○Agree |
| ○ Indeterminacy |
| ○Against |
| ○Very against |

11. I am aware that the trajectory of intrinsic capacity is influenced by factors such as lifestyle, and that there is a complex and multidirectional relationship between cognitive impairment, falls, mobility disorders, and polypharmacy and intrinsic capacity [Single choice] *

| ○Very agree |
| --- |
| ○Agree |
| ○ Indeterminacy |
| ○Against |
| ○Very against |

12. I am aware that disease and management, exercise interventions and nutritional interventions in older patients, cognitive interventions, psychosocial support and integrated multidisciplinary interventions are also important for the management of intrinsic capacity [Single choice] *

| ○Very agree |
| --- |
| ○Agree |
| ○ Indeterminacy |
| ○Against |
| ○Very against |

13. I am aware that maintenance and reversal of intrinsic competence in older patients requires further comprehensive geriatric assessment and seeking multidisciplinary collaborative interventions. [Single choice] *

| ○Very agree |
| --- |
| ○Agree |
| ○ Indeterminacy |
| ○Against |
| ○Very against |

14. Intrinsic competence is an inevitable consequence of older patients and shows a constant decline with age. [Single choice] *

| ○Very agree |
| --- |
| ○Agree |
| ○ Indeterminacy |
| ○Against |
| ○Very against |

| ○Very agree |
| --- |
| ○Agree |
| ○ Indeterminacy |
| ○Against |
| ○Very against |

15. intrinsic capacity increases rates of re-morbidity, unplanned re - hospitalization, increases patient morbidity, and decreases patient quality of life. [Single choice] *

16. Intrinsic capacity is the basis for functioning in older persons and refers to the sum of all the physical and mental capacities of an individual. [Single choice] *

| ○Very agree |
| --- |
| ○Agree |
| ○ Indeterminacy |
| ○Against |
| ○Very against |

17. Which are the main dimensions of intrinsic competence. [Multiple choice] *

| □cognitively |
| --- |
| □psychosocial |
| □hearing |
| □vision |
| □vigor |
| □campaigns |
| □currently unknown |

18. Early identification, intervention, and prevention of the progression of intrinsic capacity improves the quality of life of elderly patients and produces significant benefits to the elderly patient, family, and society. [Single choice] *

| ○Very agree |
| --- |
| ○Agree |
| ○ Indeterminacy |
| ○Against |
| ○Very against |

19.The decline in intrinsic capacity is associated with which of the following factors. [multiple choice] *

| □ Personal blood type |
| --- |
| □Type of work |
| □(a person's) age |
| □distinguishing between the sexes |
| □marital status |
| □educational attainment |
| □health behavior |
| □ Number of co-morbidities |
| □social security (pensions, medical insurance) |
| □ Residency |
| □dystrophy |
| □ inconclusive |

20.Early prompting of rational nutrition, exercise, and chronic disease management in older patients is an effective intervention to prevent or reduce intrinsic capacity in older patients. [Single choice] *

| ○Very agree |
| --- |
| ○Agree |
| ○ Indeterminacy |
| ○Against |
| ○Very against |

Part II

II. Attitudinal dimension

1. Do you think that the decline in intrinsic competence can be reversed or slowed down if health care workers pay enough attention to it [Single choice] *

| ○Very agree |
| --- |
| ○Agree |
| ○ Indeterminacy |
| ○Against |
| ○Very against |

2. Do you think that improving the state of intrinsic competence of elderly patients requires action by health care professionals [Single choice] *

| ○Very agree |
| --- |
| ○Agree |
| ○ Indeterminacy |
| ○Against |
| ○Very against |

3. you believe that it is important for healthcare professionals to maintain, halt, and even reverse the decline in intrinsic capacity for disease control and quality of life for older patients. [Single choice] *

| ○Very agree |
| --- |
| ○Agree |
| ○ Indeterminacy |
| ○Against |
| ○Very against |

4. you believe that healthcare professionals should dynamically observe the state of intrinsic competence of elderly patients. [Single choice] *

| ○Very agree |
| --- |
| ○Agree |
| ○ Indeterminacy |
| ○Against |
| ○Very against |

5. you believe that health care workers should receive formal training in knowledge of intrinsic geriatric competencies. [Single choice] *

| ○Very agree |
| --- |
| ○Agree |
| ○ Indeterminacy |
| ○Against |
| ○Very against |

6. You believe that healthcare professionals should implement a multidisciplinary intervention program (especially combining exercise and nutrition) for older patients with intrinsically diminished capacity [Single choice] *

| ○Very agree |
| --- |
| ○Agree |
| ○ Indeterminacy |
| ○Against |
| ○Very against |

7. you believe that healthcare professionals should take on the nursing role of assessing the intrinsic abilities of elderly patients. [Single choice] *

| ○Very agree |
| --- |
| ○Agree |
| ○ Indeterminacy |
| ○Against |
| ○Very against |

8. you believe that early functional exercise is important for the maintenance and reversal of intrinsic abilities in old age. [Single choice] *

| ○Very agree |
| --- |
| ○Agree |
| ○ Indeterminacy |
| ○Against |
| ○Very against |

| ○Very agree |
| --- |
| ○Agree |
| ○ Indeterminacy |
| ○Against |
| ○Very against |

9. you believe that healthcare professionals should pay as much attention to the prevention of intrinsic decline in capacity in old age as they do to other symptoms (e.g., DVT). [Single choice] *

10.You believe that patients or families need to be educated about the intrinsic capabilities of elderly patients in their clinical work. [Single choice] *

| ○Very agree |
| --- |
| ○Agree |
| ○ Indeterminacy |
| ○Against |
| ○Very against |

11.You believe that the intrinsic competence status of elderly patients should be included in the handover of clinical work. [Single choice] *

| ○Very agree |
| --- |
| ○Agree |
| ○ Indeterminacy |
| ○Against |
| ○Very against |

Part II

III. Practical dimensions

1. Do you proactively focus on the status of intrinsic abilities of older patients in your clinical practice? [Single choice] *

| ○Always |
| --- |
| ○Occasionally |
| ○Generally |
| ○Rarely |
| ○Never |

2. You would consider further multidisciplinary teamwork in the comprehensive assessment of older patients with intrinsically diminished capacity [Single choice]

| ○Never |
| --- |
| ○Rarely |
| ○Generally |
| ○Occasionally |
| ○Always |

3. do you assess the intrinsic capacity of older patients in your clinical practice? [Single choice] *

| ○Always |
| --- |
| ○Occasionally |
| ○Generally |
| ○Rarely |
| ○Never |

4. do you keep up to date with and assess your elderly patient's muscle tone status? [Single choice] *

| ○Always |
| --- |
| ○Occasionally |
| ○Generally |
| ○Rarely |
| ○Never |

5. do you keep up to date with and assess the cognitive status of your elderly patients? [Single choice] *

| ○Always |
| --- |
| ○Occasionally |
| ○Generally |
| ○Rarely |
| ○Never |

| ○Always |
| --- |
| ○Occasionally |
| ○Generally |
| ○Rarely |
| ○Never |

6. do you keep up to date with and assess the vision status of your elderly patients? [Single choice] *

7. do you keep up to date with and assess the hearing status of your elderly patients? [Single choice] *

| ○Always |
| --- |
| ○Occasionally |
| ○Generally |
| ○Rarely |
| ○Never |

8. Do you promptly attend to and assess the psychological status of your elderly patients? [Single choice] *

| ○Always |
| --- |
| ○Occasionally |
| ○Generally |
| ○Rarely |
| ○Never |

| ○Always |
| --- |
| ○Occasionally |
| ○Generally |
| ○Rarely |
| ○Never |

9. do you provide effective early functional exercise instruction for older patients? [Single choice] *

10. do you instruct family members to help the patient perform appropriate activities to relieve symptoms such as somatic weakness? [Single choice] *

| ○Always |
| --- |
| ○Occasionally |
| ○Generally |
| ○Rarely |
| ○Never |

11. do you evaluate patients in a timely manner after nursing interventions for early activity? [Single choice] *

| ○Always |
| --- |
| ○Occasionally |
| ○Generally |
| ○Rarely |
| ○Never |

12. do you evaluate your patients in a timely manner after implementing nutritional interventions? [Single choice] *

| ○Always |
| --- |
| ○Occasionally |
| ○Generally |
| ○Rarely |
| ○Never |

13. You would instruct older patients with intrinsically diminished capacity on evidence-based interventions [Single choice] *

| ○Never |
| --- |
| ○Rarely |
| ○Generally |
| ○Occasionally |
| ○Always |

14. You would provide health education or training related to intrinsic capacity to elderly patients, family members/primary caregivers with reduced intrinsic capacity [Single choice]

| ○Never |
| --- |
| ○Rarely |
| ○Generally |
| ○Occasionally |
| ○Always |

15. I will share assessments or knowledge related to intrinsic competence with other health providers [Single choice] *

| ○Never |
| --- |
| ○Rarely |
| ○Generally |
| ○Occasionally |
| ○Always |

16. Do you engage in the accumulation of knowledge related to intrinsic competence in the course of your work? [Single choice] *

| ○Always |
| --- |
| ○Occasionally |
| ○Generally |
| ○Rarely |
| ○Never |
